# Mitigating Strength Loss in Geopolymers in Low-Temperature Environments by Sodium Nitrite Addition

**DOI:** 10.3390/ma18173987

**Published:** 2025-08-26

**Authors:** Andrie Harmaji, Reza Jafari

**Affiliations:** Department of Applied Sciences, University of Québec in Chicoutimi (UQAC), 555, Boul. de l’Université, Saguenay, QC G7H 2B1, Canada

**Keywords:** geopolymer, fly ash, waste glass, bauxite residue, sodium nitrite, compressive strength

## Abstract

Geopolymer binders are a promising low-carbon substitute for Portland cement, but their behavior in cold climates remains underexplored. This study investigates the influence of sodium nitrite (NaNO_2_) on geopolymer properties cured at −10 °C for 28 days. The binders were formulated from bauxite residue, fly ash, and waste glass, and NaNO_2_ was added in various dosages as a chemical admixture. The geopolymer was tested for its setting time, compressive strength, and chemical and morphological characterizations. The addition of the 3 wt% NaNO_2_ significantly improved the strength retention in the cold environment, with a compressive strength of 40.7 MPa, compared to a geopolymer without an admixture (26.1 MPa). The X-ray diffraction (XRD) analysis confirmed the presence of gismondine, quartz, and FeSiO_3_, with NaNO_2_ remaining largely unreacted within the matrix. Fourier Transform Infrared Spectroscopy (FTIR) indicated the presence of Si–O–T bonds in the NaNO_2_-modified samples, which showed continued geopolymerization at low temperatures. Scanning electron microscopy (SEM) revealed reduced cracking and a denser microstructure with increasing concentrations of NaNO_2_. The results indicate that NaNO_2_ not only mitigates the adverse effects of subzero curing but also promotes structure development, and hence it is a viable admixture for enhancing the cold weather durability of geopolymer materials.

## 1. Introduction

Concrete structures in cold climates can deteriorate in subzero conditions, primarily due to the freeze-related expansion of water in their pore system, resulting in internal stress, cracking, and the deterioration of the structure over time [1]. Usually, concrete made with ordinary Portland cement (OPC) is particularly vulnerable to freeze–thaw damage unless it includes the addition of chemical products or other protective mechanisms [2]. Additionally, the manufacture of OPC has a large environmental footprint, representing approximately 8% of global CO_2_ emissions [3].

Due to increasing concerns over the durability and long-term sustainability of OPC-based concrete, geopolymers have gained attention as a new class of alternative binders [4]. Geopolymers are produced by the alkali activation of aluminosilicate-rich materials, such as fly ash, bauxite residue, and waste glass, which can recycle industrial waste and have lower carbon emissions compared to OPC [5,6]. Despite the advantages, there has not been considerable research on the performance of geopolymers in freezing curing conditions in terms of retaining their long-term compressive strength in freezing conditions.

Freezing can disrupt the geopolymer matrix by increasing pore pressure and causing microstructural damage, which may be intensified in mixes with increased porosity or incomplete geopolymerization [7]. While thermal curing has been shown to accelerate early strength development, it does not ensure durability under prolonged cold exposure. As a result, improving the freezing resistance of geopolymers is critical for their application in cold climates.

In previous studies, geopolymers were designed to mitigate effects of low temperatures on freeze–thaw conditions by primarily focusing on the use of air-entraining agents [8], fiber reinforcement [9], or one-part geopolymers [10] to enhance durability. However, the freeze–thaw test is a cyclic evaluation regime rather than continuous subzero curing, and while there is research on the influence of water contents on properties of alkali-activated mortars [11], studies on chemical admixtures specifically targeting early geopolymerization under cold conditions remain underexplored.

Sodium nitrite (NaNO_2_), commonly used in OPC systems as a set accelerator [12] and corrosion inhibitor [13], has shown the potential to influence early-age reaction kinetics and alter pore characteristics in cementitious materials [13]. Previous studies have reported that NaNO_2_ additions can reduce the compressive strength in both Portland cement-based concrete [13] and geopolymers [14]. Conversely, the role of NaNO_2_ as a chemical admixture of geopolymers in negative temperature curing is still unknown. To address this research gap, the present study introduces NaNO_2_ into a geopolymer matrix composed of fly ash, bauxite residue, and waste glass to evaluate its effect on preserving compressive strength under subzero conditions. Specimens were cured at 80 °C for 24 h to promote initial geopolymerization, followed by exposure at −10 °C for 28 days. The influence of NaNO_2_ on the physical properties, mechanical performance, chemical structure, and morphology of the geopolymer was investigated to determine its viability as a cold weather admixture.

## 2. Materials and Methods

### 2.1. Materials

**Figure 1:**
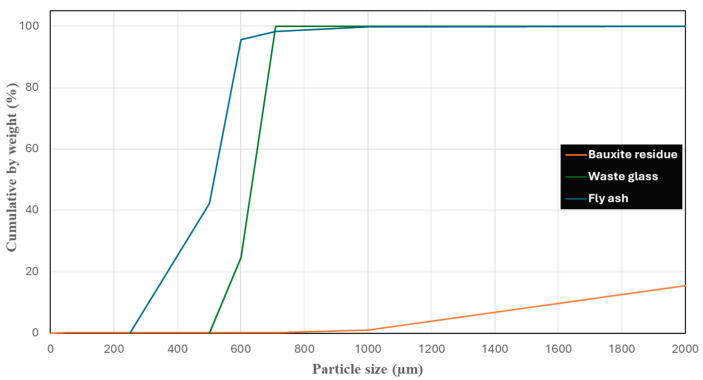
Particle size of raw materials: bauxite residue, waste glass, and fly ash.

Bauxite residue was sourced from Rio Tinto’s Jonquière complex in Saguenay, QC, Canada, and used as received without further processing. The Class F fly ash used in this study was obtained from the Shand power plant, located in Calgary, Canada. The shale is classified as Class F fly ash by the American Society for Testing and Materials (ASTM C618), where fly ash has >70 wt% total SiO_2_, Al_2_O_3_, and Fe_2_O_3_ and <18 wt% CaO. Powdered waste glass (Mirapaint X12) was sourced from the sorting facility in Bois-des-Filion, QC, Canada. The particle size of each raw material was measured by WS Tyler’s RO-Tap sieve shaker instrument, and their distribution is graphically illustrated in Figure 1. Meanwhile, their chemical compositions were analyzed by X-ray fluorescence (XRF) spectrometry using the XF700 method by Bureau Veritas Laboratories, Saint-Laurent, QC, Canada, and are presented in Table 1.

Sodium hydroxide (NaOH) pellets (77.5% Na_2_O, 22.5% H_2_O) were purchased from Laboratoire MAT (Québec City, QC, Canada) and dissolved in tap water to prepare a 16 mol/L solution. Sodium silicate solution (Na_2_SiO_3_), containing 8.9% Na_2_O, 28.7% SiO_2_, and 62.4% H_2_O, was supplied by an industrial supplier in Richmond, Canada. The solutions were thoroughly mixed and allowed to rest for 6 h at room temperature prior to use to minimize exothermic reactions during mixing, which can cause efflorescence [15]. Natural sand with a fineness of 80 µm was sourced from a quarry in Saint-Henri-de-Taillon, Canada, and used in both the geopolymer and OPC mortar formulations. Its fineness modulus and absorption capacity were 2.9 and 0.3%, respectively. Sodium nitrite (NaNO_2_) with 97% purity, used as the antifreeze chemical admixture, was acquired from Thermo Scientific, Ottawa, ON, Canada.

### 2.2. Mix Design Proportions

Five mixtures were formulated to investigate the influence of NaNO_2_ addition on geopolymer strength retention in subzero conditions. The geopolymer binders were made from a combination of bauxite residue, fly ash, and waste glass. Alkali activator solution was made by mixing NaOH 16 mol/L and liquid Na_2_SiO_3_ (8.9% Na_2_O, 28.7% SiO_2_, and 62.4% H_2_O) in a 2:1 weight ratio. The binder materials and natural sand were mixed thoroughly in a planetary mixer before the gradual addition of an alkali activator. The binder-to-liquid ratio was fixed at 0.45. NaNO_2_ was added directly to slurry mixture at combinations of 0–3 wt% of geopolymer binders, with the mixer continuing to operate until a consistent slurry was produced. The complete mixing proportions are found in Table 2.

The fresh mortar was poured into a 50 mm × 50 mm × 50 mm mold according to ASTM C109 and sealed with a hydrophobic film to minimize moisture evaporation during curing. The mortar specimens were thermally cured at 80 °C for 24 h in a laboratory oven to facilitate the geopolymerization [16]. After demolding, C samples were exposed to ambient conditions for 7 and 28 days prior to testing. Meanwhile, the N0, N1, N2, and N3 samples were stored at −10 °C for similar times to evaluate their mechanical properties under negative temperature exposure.

### 2.3. Setting Time Test

The setting time of the geopolymer mixtures was evaluated following the procedure outlined in ASTM C191 using the Vicat apparatus. For this purpose, paste mixtures from the same binder composition as the corresponding mortar formulations were prepared, without the addition of aggregates to eliminate the influence of particle interlock on setting behavior. Immediately after mixing, the newly mixed paste was poured into a standard Vicat mold in truncated conical form with an internal diameter of 70 ± 5 mm at the top and 80 ± 5 mm at the bottom and a height of 40 ± 0.2 mm, resting on a non-absorbent base glass plate.

The mold was filled in a single lift and leveled to ensure an equal thickness from the base to the superior surface. The paste specimens were maintained under the same ambient curing conditions as the mortars to ensure consistency between the two tests. Penetration resistance measurements were recorded at predetermined time intervals using the Vicat needle, and the initial setting time was defined as the elapsed time from initial contact between binder and activator until the needle penetration reached 25 ± 1 mm. The final setting time was determined when the needle failed to visibly penetrate the paste surface. This method enabled a direct assessment of the intrinsic setting characteristics of the binder system, without the confounding effects of aggregate gradation on surface area.

### 2.4. Compressive Test Methods

Compressive tests were conducted after 7 and 28 days of curing to evaluate long-term strength. The tests were carried out using a PILOT PRO—EN Automatic Compression Tester machine in Saguenay, QC, Canada applying a uniaxial compressive load at a controlled rate of 0.25 ± 0.05 MPa/s until specimen failure. The reported compressive strength values represent the average of at least three specimens per mixture.

### 2.5. XRD Analysis

**Figure 2:**
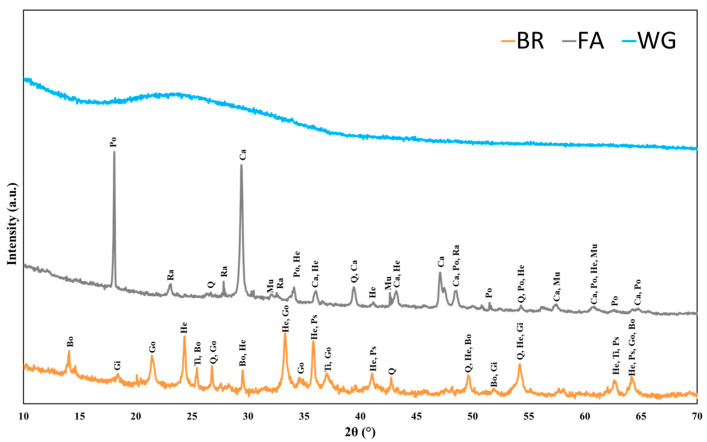
X-ray diffraction (XRD) patterns of the raw materials [Q: quartz (JCPDS 05-0490), He: Hematite (JCPDS 06-0502), Mu: Mullite (JCPDS 02-0428), Gi: Gibbsite (JCPDS 03-0145), Go: Goethite (JCPDS 02-0281), Bo: Boehmite (JCPDS 03-0065), Po: Portlandite (JCPDS 02-0969), Ca: Calcite (JCPDS 02-0623), Ra: Rankinite (JCPDS 11-0317), Ti: Anatase (JCPDS 02-0406), and Ps: Pseudorutile (JCPDS 19-0635)].

The X-ray diffraction (XRD) method was used to analyze the chemical composition of raw materials (wet filter-pressed bauxite residue, fly ash, and waste glass) and hardened geopolymers. The analysis was performed using a Panalytical X’Pert Pro MPD diffractometer in Sherbrooke, QC, Canada, scanning from 5° to 80° (2θ) at a rate of 2.5°/min over a total acquisition time of 30 min. Then, the diffractogram was analyzed using XPowder ver. 2004.04.70 Pro software and cross-referenced with the Joint Committee on Powder Diffraction Standards (JCPDS) database to identify the crystalline phases present. The primary goal was to examine the crystalline and amorphous phases in geopolymer pastes to determine the effect of NaNO_2_ content and curing method on geopolymer mineralogy. The samples for XRD investigations included the raw materials and debris from the compression strength specimens, which were ground using a mortar and pestle to a fine powder suitable for placement in the sample holder. X-ray diffraction patterns of the raw materials are depicted in Figure 2. The wet filter-pressed bauxite residue indicates boehmite (Al_2_O_3_H_2_O), gibbsite (Al(OH)_3_), goethite (FeO(OH)), anatase (TiO_2_), pseudorutile (Fe_2_Ti_3_O_9_), quartz (SiO_2_), and hematite (Fe_2_O_3_) as the main crystalline phases. Subsequently, fly ash showed the appearances of mullite (3Al_2_O_3_2SiO_2_), calcite (CaCO_3_), portlandite (Ca(OH)_2_), and rankinite (Ca_3_Si_2_O_7_) alongside quartz and hematite. Meanwhile, waste glass showed amorphous diffractogram, while the broad halo demonstrates compounds of amorphous silica phases between 20° and 37°, characteristically found in structurally disordered compounds [17].

### 2.6. FTIR Characterization

Fourier Transform Infrared Spectroscopy (FTIR) analyses were performed in attenuated total reflectance (ATR) mode utilizing a Cary 630 FTIR spectrometer (Agilent, Santa Clara, CA, USA). Spectra were collected over a range of 4000–400 cm^−1^ with a spectral resolution of 2 cm^−1^. Each spectrum represented the average of 64 scans to increase the signal-to-noise ratio. The data were processed and analyzed using PerkinElmer Spectrum 10 spectroscopy software for peak identification and band assignment.

### 2.7. Morphological Analysis

The microstructural and morphological characteristics of the geopolymer specimens were examined using a JEOL JSM-6480HV (Tokyo, Japan) scanning electron microscope (SEM). Cross-sectional slices of the specimens were prepared from 28-day-cured samples, ensuring representative regions for imaging. Prior to analysis, the specimen surfaces were dried and mounted on aluminum stubs using conductive carbon tape. The SEM was operated under high-vacuum mode at 12 mm working distance, and images were captured at 500× and 1000× magnifications to observe the morphology of the geopolymer matrix, unreacted particles, pore structures, and the interface between reaction products and aggregates.

## 3. Results and Discussion

### 3.1. Setting Time

**Figure 3:**
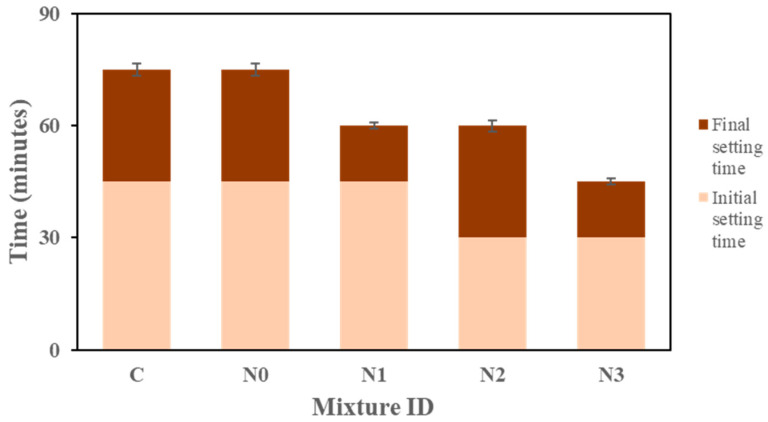
The impact of the NaNO_2_ concentration on the setting times of the geopolymers.

The setting times of the geopolymers were measured to evaluate the influence of NaNO_2_ on the early-age behavior of the fresh mixtures. The results for initial and final setting times are presented in Figure 3. The control (C) and the geopolymer without NaNO_2_ cured under negative temperatures (N0) both exhibited identical physical properties, with an initial setting time of 45 min and a final setting time of 75 min. The addition of NaNO_2_ significantly affected the setting behavior. At a low dosage (N1), the initial setting time remained unchanged from the control but achieved a slightly faster final setting (60 min), indicating that minor amounts of NaNO_2_ do not accelerate the reaction significantly.

Further increases in the NaNO_2_ percentage (N2 and N3) caused noticeable acceleration. N2 recorded initial and final setting times of 30 and 60 min, while N3 exhibited the fastest, achieving the initial and final setting in just 30 and 45 min, respectively. These results shows that NaNO_2_ works as a setting accelerator in the geopolymer system, likely as a result of higher concentrations of sodium cations (Na^+^) enhancing the dissolution of aluminosilicate species [18]. The acceleration is dose-dependent and becomes more significant at higher NaNO_2_ contents. Rapid setting, while beneficial for early strength gain [19], must be carefully managed to avoid issues such as reduced workability [20] or increased shrinkage [21]. Xu et al. [22] further noted that this swift behavior offers a major advantage for winter rapid-repair purposes. Hence, incorporating NaNO_2_ aligns well with the cold temperature challenges highlighted in this study.

### 3.2. Compressive Strength

**Figure 4:**
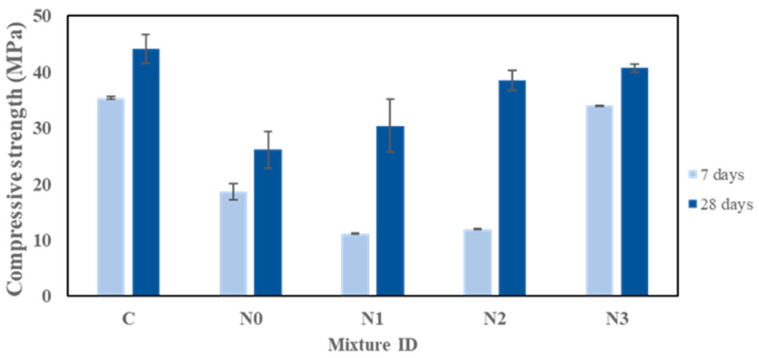
The effect of the NaNO_2_ concentration on the compressive strength of the geopolymers.

Figure 4 presents the compressive test data for five samples (C, N0, N1, N2, and N3) at 7 and 28 days of curing. The results demonstrate varying rates of strength development across the different samples.

Sample C, the control geopolymer in the ambient temperature curing, exhibited a steady strength gain from 35.4 ± 0.26 MPa to 44.1 ± 2.62 MPa. The moderate reaction rate under the ambient curing likely enabled continuous geopolymer gel formation, resulting in a dense microstructure and stable strength development, consistent with prior reports on room-temperature-cured alkali-activated slag/bauxite residue systems [23].

Sample N0, which did not contain NaNO_2_ but was subjected to subzero curing, achieved a lower early strength (18.6 ± 1.5 MPa at 7 days) and a modest increase to 26.1 ± 3.2 MPa at 28 days. The reduced initial strength and slower progression of the strength gain are due to the freezing, which provides an inhibitory effect on dissolution–polycondensation reactions that occur, which slows the formation of the gel during the early curing [24]. Similar delays in strength development at low temperatures have been observed for both the OPC and geopolymer matrix, where reaction kinetics drop sharply below 10 °C due to reduced ion mobility, resulting in a less dense and porous microstructure [25]. Sample N1 demonstrated a significant increase in strength, from 11.2 ± 0.1 MPa at 7 days to 30.4 ± 4.8 MPa at 28 days. Meanwhile, sample N2 exhibited an average of 38.5 MPa at 28 days. At the same age, sample N3 achieved 40.7 ± 0.7 MPa under similar curing conditions. This result shows effective strength retention under the negative temperature environment, improving the negative temperature’s durability of materials by enabling matrix densification and continued reactions. Sodium nitrite acts as a freezing-point depressant and speeds up the dissolution of aluminosilicate precursors, which means the geopolymerization continues to occur in subzero curing conditions. More sodium aluminosilicate hydrate (N-A-S-H) gels are produced to fill pores and densify the matrix. A denser microstructure reduces the extent of the microcracking caused by ice formation [26], therefore providing improved durability. In other alkali-activated systems, sodium nitrite has been studied as well by Ma et al. [27]. The research found that in sulphoaluminate cement, sodium nitrite greatly improved the low-temperature performance by accelerating the early hydration and decreasing frost damage. Similarly, the main benefit of NaNO_2_ in this study lies in preserving the strength of the geopolymer rather than promoting additional gains, making it a valuable additive for maintaining performances in cold environments.

### 3.3. XRD Characterization

**Figure 5:**
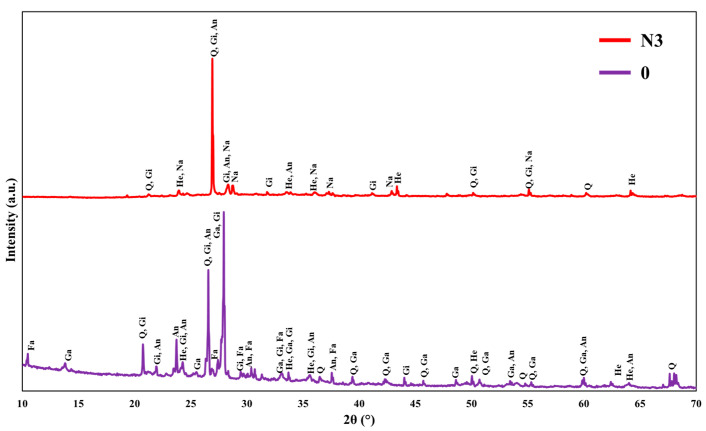
XRD diffractograms of geopolymer samples: without admixture (C) and N3 (3 wt% NaNO_2_) after −10 °C curing for 28 days [Q: quartz (JCPDS 05-0490), He: hematite (JCPDS 06-0502), Fa: faujasite (JCPDS 11-0672), Ga: gaylussite (JCPDS 02-0528), An: anorthite (JCPDS 02-0537), Gi: gismondine (JCPDS 20-0452), and Na: sodium aluminum amide (JCPDS 20-1068)].

Figure 5 displays the XRD patterns of the geopolymer without an admixture (C) and with the 3 wt% NaNO_2_ addition (N3) after 28 days of curing in subzero temperatures. The XRD patterns revealed the presence of amorphous and crystalline phases; the former was attributed to either (a) the phase from the raw materials [27] or (b) a newly produced amorphous phase caused by geopolymerization, as demonstrated by the gel product that typically formed within four hours [28]. This dominant amorphous nature of the control sample is a characteristic of alkali-activated material systems and is often associated with improved density [29] and thermal stability [30].

The XRD pattern of sample C revealed the presence of several crystalline and amorphous phases. Among the detected crystalline peaks is hematite (Fe_2_O_3_), which is attributed to unreacted iron oxide remnants from the bauxite residue [31]. This was already confirmed by the XRF of the bauxite residue (Table 1), which revealed high amounts of Fe_2_O_3_ (44.4%), and the XRD diffractogram from Figure 2. The presence of quartz (SiO_2_) could be attributed to unreacted sand particles or crystalline silica from the raw materials [32]. The presence of anorthite (Al_2_Ca(SiO_4_)_2_) suggests that calcium from the waste glass reacted with accessible aluminosilicate phases throughout the curing, resulting in a solid crystalline phase. Its existence implies enough Ca availability to improve the geopolymer matrix’s long-term mechanical performance [33]. A peak assigned to gaylussite (Na_2_CO_3_·CaCO_3_·5H_2_O) was also observed. This phase in alkali-activated materials could be formed during the exposure to ambient conditions after the oven curing, indicating a reaction between free Na_2_O and atmospheric CO_2_, resulting in a carbonation product often seen in systems with high sodium contents and calcium-rich binders [34]. Another study leveraged the beneficial effects of sodium bicarbonate (Na_2_CO_3_) by using it as an activating agent in geopolymer mixtures containing local zeolite and slag, resulting in improved flexural strength [35]. Interestingly, gismondine (CaAl_2_Si_2_O_8_·4H_2_O) was identified as a major aluminosilicate phase. The formation of this compound may be promoted by the relatively high CaO content originating from the waste glass, in contrast to the typical formation of albite species as a geopolymerization product of NASH gel [36]. This zeolitic phase acts as a stable crystalline product that promotes the growth of the aluminosilicate network, which is known to improve geopolymerization [37]. Another crystalline zeolite-type phase identified is faujasite (Na_2_Al_2_Si_4.7_O_13.4_xH_2_O), likely originating from the crystallization of N-A-S-H gels during the oven curing. Under elevated curing temperatures, the amorphous geopolymer gel can partially rearrange into more thermodynamically stable zeolitic phases, a phenomenon often observed in low-calcium alkali-activated binder systems [33].

In the N3 diffractogram, the pattern exhibits a more pronounced amorphous hump, with only a few minor peaks corresponding to crystalline phases. The peaks related to faujasite have disappeared, indicating an increased degree of geopolymerization compared to the control [38]. Moreover, this sample retained the presence of gismondine, quartz, and hematite phases as in the control sample without the admixture. The persistent presence of gismondine further confirms the substantial formation of geopolymerization products within the matrix with a Ca-rich character and corresponds with the contribution of CaO introduced from the waste glass. Furthermore, a relevant phase was found to be sodium aluminum amide (NaAl(NH_2_)_4_), which presumably formed via the interaction of aluminum species originating from the binder and sodium-bearing and nitrogen-rich compounds, under the subzero curing. The presence of sodium aluminum amide suggests that low-temperature curing may have been beneficial in stabilizing amide complexes, possibly because hydrolysis was inhibited and/or the geopolymerization hydrolysis pathways were altered. While it is not clear what role sodium aluminum amide plays in mechanical performance, its mere presence indicates that there were potentially different chemical environments and bonding configurations under freezing conditions. Intriguingly, gaylussite phases were absent in this sample. This can be attributed to the lack of a direct interaction with atmospheric CO_2_ under freezing conditions. Since the samples were stored at –10 °C, carbonation reactions were likely suppressed due to the reduced CO_2_ diffusion and the absence of the ambient curing exposure. The freezing temperatures probably immobilized the pore solution and limited the available free water to be used for crystallization purposes. As the temperature dropped, water began to freeze in the capillary pores, forcing the free water and remaining water into larger diameter pores [39]. A slight shift in peak positions between C and N3 is normal and can be attributed to minor variations in the instrument calibration and sample alignment or differences in the lattice strain resulting from the incorporation of NaNO_2_ and its influence on the geopolymer gel network. Such small shifts are commonly observed in XRD measurements and do not indicate a significant change in the presence of crystalline phases.

### 3.4. FTIR Analysis

**Figure 6:**
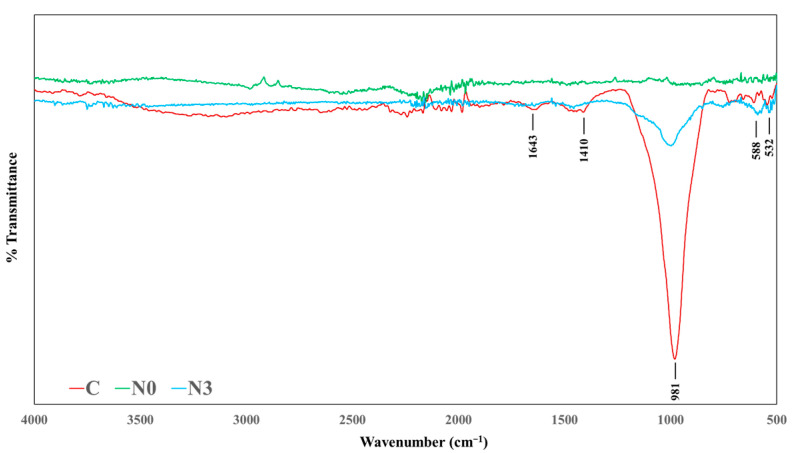
Fourier transform infrared (FTIR) spectra of C (without admixture), N0 (0 wt% NaNO_2_), and N3 (3 wt% NaNO_2_). Samples N0 and N3 were cured in negative temperatures (−10 °C) for 28 days.

To determine the chemical composition of the geopolymers in normal and negative temperature curing, the cross-section of samples C, N0, and N3 after 28 days were analyzed by using FTIR (Figure 6). The region beneath the band at ~1000 cm^−1^ contains stretching vibrations in Si-O-T, where T represents tetrahedrally bound Al or Si, indicating the geopolymerization process [40]. The spectrum’s transmittance was significantly higher for the geopolymer samples in the ambient curing.

The ambient-cured control sample (C) showed a band at ~532 cm^−1^, corresponding to Fe–O bands [41,42], which could be attributed to the hematite phase. A sharp and well-defined absorption band was observed at ~981 cm^−1^, corresponding to the Si–O–T bonds [43]. However, the N0 sample, which was subjected to −10 °C curing for 28 days without the NaNO_2_ addition, exhibited a significantly diminished or nearly absent Si–O–T peak, suggesting incomplete geopolymerization and a weakened aluminosilicate framework. The disappearance of this peak, which could also be proof of anorthite d, gives us direct empirical evidence from our theory that freezing conditions destabilize the Si–O–T network and promote the crystallization of secondary phases. The loss of the Si–O–T bond might be linked with anorthite formation, suggesting that the destruction of the aluminosilicate network due to freezing conditions favored the crystallization of this secondary phase. Conversely, the N3 sample, which also underwent the negative temperature curing conditions, retained some portion of the Si–O–T band, albeit with a reduced intensity compared to the ambient-cured control. These preservation of the Si–O–T bonding is consistent with the inhibition of anorthite crystallization and the formation NaAl(NH_2_)_4_, providing direct evidence that NaNO_2_ helped maintain the connectivity of the network and inhibited unwanted phase changes during the curing below 0 °C. The preserved structural signature in N3 aligns with its higher 28-day strength (40.7 MPa), compared to N0 (26.12 MPa), and nearly approaches that of the ambient-cured control (44.18 MPa). This result further supports the role of NaNO_2_ in maintaining the structural integrity under cold curing. Additionally, a new absorption band observed at ~588 cm^−1^ in N3, which was not strongly visible in C or N0, can be attributed to the T-O peak [44]. This is a characteristic of well-developed aluminosilicate frameworks and polymerized products [45], identified as gismondine in the XRD analysis. According to Merabtene et al. [46], this band typically appears after 28 days of hardening and is associated with enhanced phase crystallization. Its emergence in N3 suggests that NaNO_2_ promotes the crystallization of secondary aluminosilicate phases, likely by facilitating ion mobility or providing favorable chemical conditions even under freezing environments. This further explains the strength preservation of the NaNO_2_-modified geopolymer.

The absorption bands at ~1410 cm^−1^ correspond to stretching vibrations of C=O [47], confirming the presence of carbonate compounds in the form of gaylussite, which are only visible in the spectra of sample C. Collectively, these findings suggest that NaNO_2_ plays a critical role in preserving the structural and chemical integrity of geopolymer networks in terms of Si–O–T bonds during the low-temperature exposure, thereby enhancing the durability and performance in cold environments. The FTIR characterization supported these results by showing that the Si–O–T stretching band present in N0 disappears after a prolonged low-temperature exposure without NaNO_2_, whereas N3 retains a distinct Si–O–T peak, indicating the partial preservation of the geopolymer network.

### 3.5. Morphological Analysis with SEM Characterization

**Figure 7:**
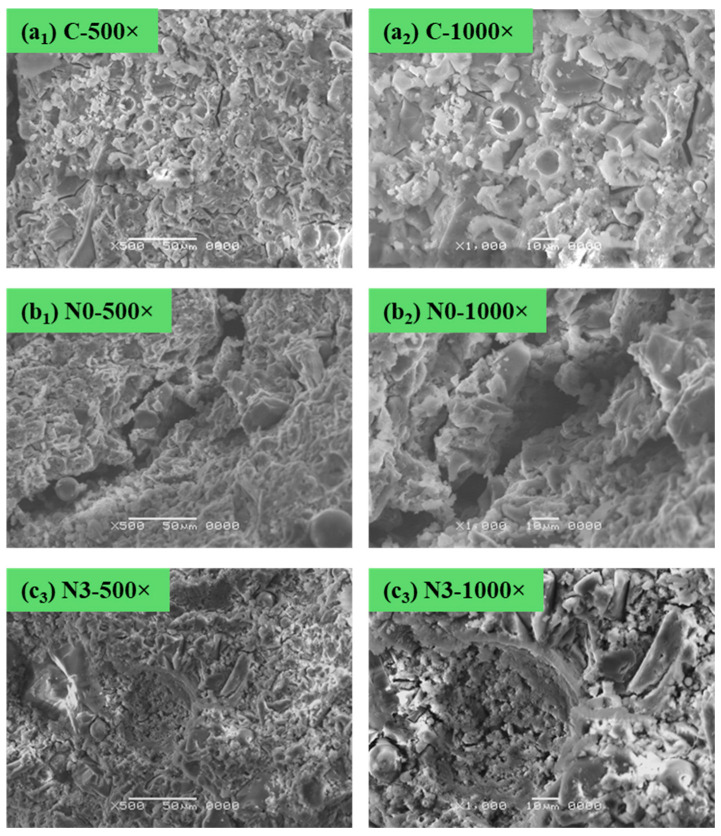
SEM images of geopolymers (**a_1_**) C magnified at 500×, (**a_2_**) C magnified at 1000×, (**b_1_**) N0 magnified at 500×, (**b_2_**) N0 magnified at 1000×, (**c_1_**) N3 magnified at 500×, and (**c_2_**) N3 magnified at 1000× after curing in different temperatures for 28 days.

SEM was used to investigate the morphology of the geopolymers at different curing temperatures. Figure 7 shows the topography of all geopolymer samples at a 500× and 1000× magnification. The SEM characterization revealed morphological differences between the geopolymer samples exposed to ambient and subzero curing for 28 days. Sample N0, where NaNO_2_ was absent, exhibited wide microcracks throughout the matrix. These cracks were likely the result of internal stress induced by the ice crystallization and thermal shrinkage during the prolonged exposure to negative temperatures, which is known to compromise the structural integrity of cementitious materials [48]. In contrast, sample N3 showed visibly narrower and more hairline-like cracks, suggesting a mitigation of cold-induced damage. These observations indicate that NaNO_2_ functions as an antifreeze admixture, mitigating internal freezing and microcracking by stabilizing the pore solution and enhancing matrix densification.

Furthermore, the SEM images revealed the partial filling of fly ash spherical cavities, which are normally observed as hollow cenospheres [49], with newly formed gel-like material. This infilling phenomenon, which was absent in sample N0, indicates that the added NaNO_2_ may facilitate continued geopolymer gel formation during cold curing, contributing to strength retention. This finding is in line with the XRD results, where distinct peaks confirmed the presence of NaNO_2_ and the aluminosilicate gel in the form of gismondine. The presence of these crystalline regions of NaNO_2_ was not only chemically reactive during geopolymerization but also retained in the matrix. This addition could enhance the matrix densification and contribute to strength preservation in freezing environments, suggesting that NaNO_2_ not only facilitates the acceleration of geopolymer setting but also stabilizes the material under extreme conditions, making it a promising additive for improving the performance of geopolymers in cold environments.

## 4. Conclusions

The effects of sodium nitrite (NaNO_2_) as a chemical admixture on the mechanical and microstructural performance of geopolymers cured under subzero conditions were systematically evaluated. The key conclusions drawn from this study are as follows:

1. The addition of 3 wt% NaNO_2_ significantly improves the compressive strength retention of geopolymers during low-temperature curing (40.7 MPa at 28 days), which is comparable to 44.2 MPa for the control samples.
2. Geopolymers without NaNO_2_ show the severe degradation of strength when cured under subzero conditions due to incomplete geopolymerization, supported by the absence of Si–O–T bonds in FTIR spectral data and the identifiable cracking in SEM images.
3. The XRD results indicate that the NaNO_2_ addition retained the stable geopolymer gel structure in gismondine, and the absence of the faujasite phase indicates the increased degree of geopolymerization. Furthermore, the formation of the new sodium aluminum amide phase could be responsible for stabilizing the performance of the geopolymer in low-temperature environments.
4. SEM observations reveal less cracks in the geopolymer containing NaNO_2_, which suggests a better microstructural integrity and resistance to fracture propagation.

## Figures and Tables

**Table 1:** The chemical composition of wet filter-pressed bauxite residue, fly ash, and waste glass.

Oxide Mass (%)	Bauxite Residue	Fly Ash	Waste Glass
**SiO_2_**	11.1	43.5	72.2
**Al_2_O_3_**	17.2	21.1	1.6
**Fe_2_O_3_**	44.4	26	0.4
**MgO**	0.05	0.7	1.1
**CaO**	2.6	3.8	11.2
**Na_2_O**	6.4	0.5	12.9
**K_2_O**	0.06	1.7	0.5
**LOI**	11.9	1.9	-

**Table 2:** The mix design of geopolymer samples with different NaNO_2_ percentages.

Name	Bauxite Residue (kg/m^3^)	Fly Ash (kg/m^3^)	Waste Glass Powder (kg/m^3^)	Fine Aggregate (kg/m^3^)	NaOH (kg/m^3^)	Na_2_SiO_3_ (kg/m^3^)	NaNO_2_ (kg/m^3^)	Curing Method
C	302.8	113.5	340.7	1514	101	202	-	ambient
N0	302.8	113.5	340.7	1514	101	202	-	subzero
N1	302.8	113.5	340.7	1514	101	202	7.6	subzero
N2	302.8	113.5	340.7	1514	101	202	15.2	subzero
N3	302.8	113.5	340.7	1514	101	202	22.8	subzero

## Data Availability

The original contributions presented in the study are included in the article, further inquiries can be directed to the corresponding author.
